# Alternative splicing in plant stress responses: potential application for crop improvement

**DOI:** 10.1007/s00425-025-04847-2

**Published:** 2025-11-01

**Authors:** Wai Keat Toh, Hann Ling Wong, Mee-Len Chye

**Affiliations:** 1 Faculty of Science, Universiti Tunku Abdul Rahman, Jalan Universiti, Bandar Barat, 31900 Kampar, Perak Malaysia; 2 Centre for Agriculture and Food Research, Faculty of Science, Universiti Tunku Abdul Rahman, Jalan Universiti, Bandar Barat, 31900 Kampar, Perak Malaysia; 3 School of Biological Sciences, The University of Hong Kong, Pokfulam, Hong Kong, China

**Keywords:** Biotic stress, Environmental stress, Exon, Intron, Intron retention, Splice variants

## Abstract

**Main conclusion:**

Alternative splicing (AS) which increases the diversity of the transcriptome frequently occurs in plants following stress treatment. Transcripts from AS offer potential for designing more resilient crops.

**Abstract:**

Rapid advances in technology on full-length transcriptome sequencing (e.g. long-read single-molecule real-time), high-throughput RNA sequencing, direct RNA-sequencing platforms and high-throughput analysis have provided rapid characterization of transcriptomes with frequent encounters of alternative splicing (AS) in plants, particularly following biotic and abiotic (heat, low temperature, drought, lead and salt) stress treatments. Comprehensive plant databases of stress-responsive AS events, including those from Arabidopsis (*Arabidopsis thaliana*) and rice (*Oryza sativa*), indicate that intron retention is most prevalent. Given that plants are sessile, AS allows the plant to diversify its transcriptomic and proteomic landscape to enhance stress protection. Sometimes, the overexpression of a splice variant in transgenic plants will result in protection against the AS-triggered stress. Recent examples of stress-related AS in plants will be discussed together with the potential of splice variants in designing more resilient crops to thwart climate change, improve productivity and enhance food security.

## Introduction

Following transcription in the plant nucleus, the precursor mRNA (pre-mRNA) transcript is subject to splicing by the spliceosome and introns are cleaved to yield mRNA. In alternative splicing (AS), variations in the mature mRNA result when specific introns in the pre-mRNA are included (intron retention) or exons excluded (exon skipping); alternative 5’ or 3’-splicing sites are used, or application of mutually exclusive exons or isoforms occurs (Marasco and Kornblihtt 2023). Multiple forms of transcripts derived from a single gene will diversify the transcriptome and enrich the proteome with AS-derived protein sequences (Doll 2024). AS occurs in ~ 95% of human and ~ 70% of plant intron-containing genes (Jabre et al. 2019). About 8% of plant AS events involve exon skipping, while 45–56% of those in Arabidopsis (*Arabidopsis thaliana*) involve intron retention (Sybilska and Daszkowska-Golec 2023). In human and mouse genomes, 77% of multi-exonic genes result from intron retention (Braunschweig et al. 2014). Advances in transcriptomics and proteomics have helped identify AS events during plant development and metabolism, as well as in response to hormones, abiotic stress and biotic stress (Liu et al. 2022; Mandadi et al. 2023).

AS regulates plant metabolism and development via the modulation of biochemical activities and subcellular protein localization (Lam et al. 2022). Pre-mRNAs of genes in both primary [e.g., starch, lipid, photorespiration, ascorbate, abscisic acid (ABA), auxin] and secondary (e.g., jasmonate, terpenoids, alkaloids and phenolpropanoids, flavonoids) metabolism are subject to AS (Lam et al. 2022). AS-regulated developmental events occur during plant development (embryogenesis, seed germination, skotomorphogenesis and photomorphogenesis), flowering, fruit ripening and wood formation (Szakonyi and Duque 2018; Xue et al. 2021; Lam et al. 2022; Yang et al. 2022; Sybilska and Daszkowska-Golec 2023).

Many alternatively-spliced genes related to signalling of the phytohormone ABA regulate ABA-associated components in seed germination (Xue et al. 2021; Sybilska and Daszkowska-Golec 2023). A component of this pathway, HYPERSENSITIVE TO ABA1 (HAB1) phosphatase belongs to the PP2C phosphatase family and AS generates isoforms HAB1.1 and HAB1.2 that antagonistically modulate seed germination (Wang et al. 2015). Germination-promoting HAB1.1 is the full-length protein in contrast to HAB1.2, which retains an intron and inhibits germination (Wang et al. 2015). The presence of a premature stop codon arising from AS deprives HAB1.2 of 105 amino acid residues at the *C*-terminus in contrast to HAB1.1 (Wang et al. 2015). Thus, HAB1.2 lacks the catalytic domain for the dephosphorylation of protein kinase SnRK2.6, a component in ABA signalling, and cannot inhibit kinase activity and interrupt ABA signalling (Wang et al. 2015). Besides being subject to AS modulation during seed germination (Sybilska and Daszkowska-Golec 2023), ABA being a signal for cold, drought and salt stress, is similarly regulated in abiotic stress responses (Yang et al. 2022). However, the role of ABA in AS regulation during stress responses and development is less clear (Yang et al. 2022). After ABA treatment of the monocot, rice (*Oryza sativa*), the spliceosome-associated FK506-binding protein OsFKB20-1b was shown to promote AS with intron retention (Jung et al. 2023).

## Alternative splicing in plants upon biotic stress

AS has been applied as a strategy to protect against phytopathogens (Lam et al. 2022). Using next-generation sequencing technology on sorghum (*Sorghum bicolor*) infected with *Colletotrichum sublineola*, the causative agent of anthracnose, a considerable number of genes of the flavonoid and phenylpropanoid biosynthetic pathways were demonstrated to be subject to AS (Wang et al. 2020). The most abundant AS events reported, alternative first or last exon ends in transcript isoforms arising from in-frame internal deletions, caused exclusion of biochemical interactive sites and interrupted protein function (Wang et al. 2020). Also, splice factors such as the spliceosome-associated serine/arginine rich (SR) proteins, which function in spliceosome assembly and splice site recognition, were subject to AS following fungal infection (Wang et al. 2020). The application of such AS-derived pathogen-inducible SR protein isoforms, altered in signal transduction or metabolic activity, could pave to new strategies in *S. bicolor* protection (Wang et al. 2020).

RNA-seq datasets from hot pepper (*Capsicum annuum*) after infection by bacteria [*Xanthomonas axonopodis* pv. *glycines* 8ra (Xag8ra), *Xanthomonas campestris* pv. *vesicatoria* race 1 (Xcv1), and *X. campestris* pv. *vesicatoria* race 3 (Xcv3)], oomycete *Phytophthora capsica* or tobacco mosaic virus P2 strain, identified differential alternatively-spliced genes responsive to these phytopathogens (Kim et al. 2024). AS events were also derived from distinct tissue types, such as root, stem, flower as well as the placenta and pericarp in fruit development, besides post treatment from abiotic stress (cold, heat and osmosis) or signalling molecules such as salicylic acid, jasmonic acid, ABA, and ethylene (Kim et al. 2024). The results yielded a sum of 1,642,007 AS events and 4354 differentially alternatively spliced genes related to environmental stressors, tissues, and signalling molecules (Kim et al. 2024). The highest number of AS events occurred after treatment from biotic stress (689,238), followed by abiotic stress (433,339), and signalling molecules (389,911) (Kim et al. 2024). Stress treatments on *Capsicum annuum* caused AS events with exon skipping with the use of an alternative 5'-splice site predominant (Kim et al. 2024). Exon skipping (88.52%) emerged to be the most common AS event following flagellin (*flg22*) treatment of the Chinese wild grape (*Vitis quinquangularis*), yielding 9156 AS events of genes related to stress resistance and splicing factors (Yao et al. 2025).

The spliceosome, where pre-mRNA splicing occurs consists of ribonucleoproteins and auxiliary proteins (Yang et al. 2022b; Marasco and Kornblihtt 2023). RNA-binding proteins are subject to arginine methylation by protein arginine methyltransferases (PRMTs; Deng et al 2010). ABA promotes arginine methylation of the spliceosome component SM-LIKE PROTEIN4 (LSM4), by enhancing PRMT activity for the transfer of the methyl groups from *S*-adenosylmethionine to the guanidino nitrogen atoms of the arginine residues within LSM4, resulting in the methylation of LSM4 (Agrofoglio et al. 2024). Arginine methylation of LSM4 is essential for AS and plant growth under abiotic stress (Agrofoglio et al. 2024). However, during bacterial infection, LSM4 methylation declined and plants harbouring unmethylated LSM4 showed better protection against *Pseudomonas* but were hypersensitive to ABA and salt stress in comparison to those expressing the wild-type LSM4 (Agrofoglio et al. 2024). As hypersensitivity to ABA and salt stress resulted when LSM4 methylation was impaired, arginine methylation of LSM4 appears to function antagonistically in the modulation of AS during abiotic and biotic stress (Agrofoglio et al. 2024).

**Fig. 1:**
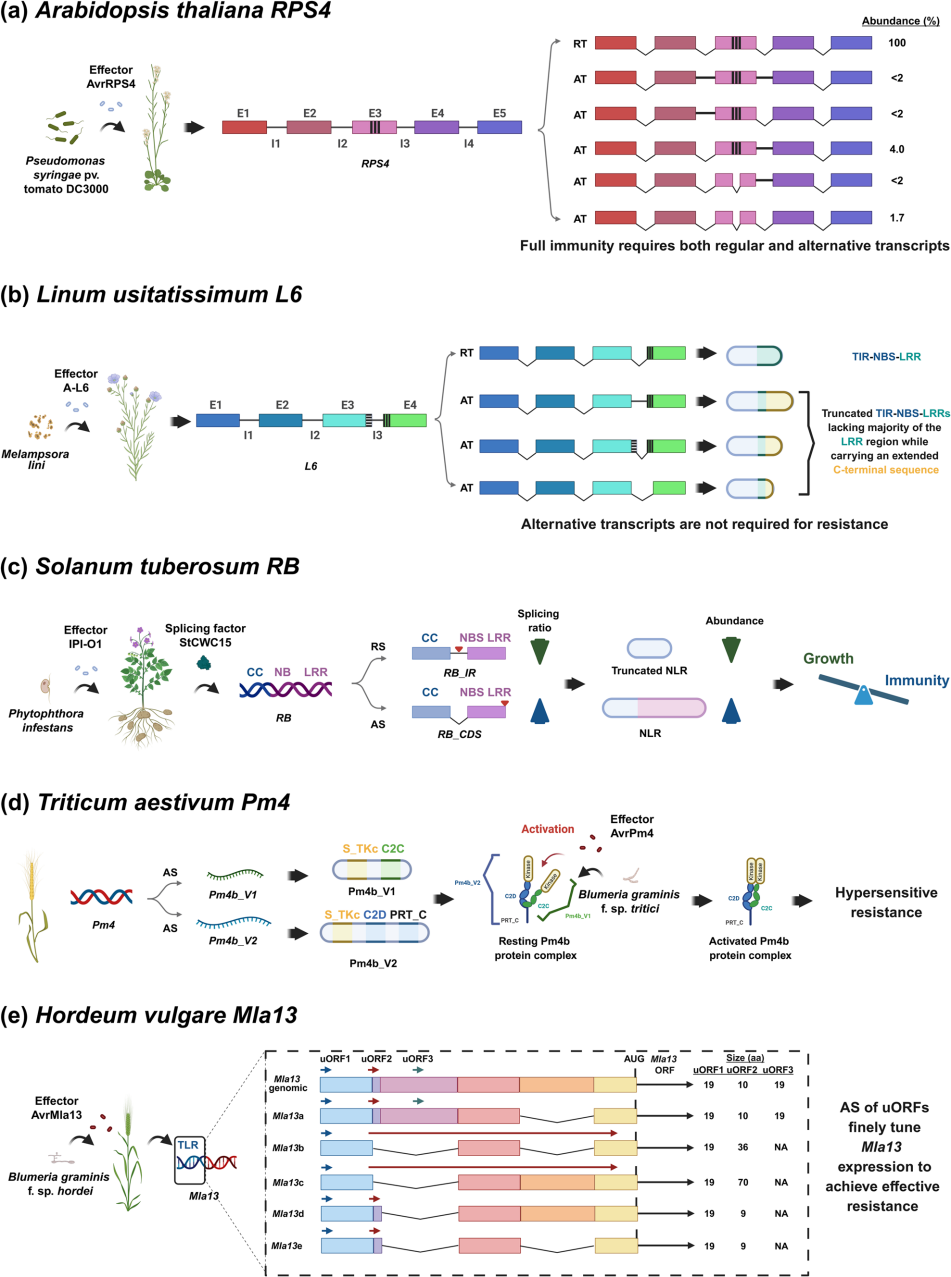
Diverse alternative splicing mechanisms underpin plant immune responses to biotic stress. AS can generate transcript diversity across genes related to plant immunity during pathogen challenges, contributing to distinct immune strategies. The spliced and retained introns are shown as angled and straight lines, respectively. The cryptic introns are indicated by vertically hatched boxes in the exons, and horizontally hatched boxes for cryptic exons in introns. E:exon; I: intron. **a**-**e** The examples span across various species and highlight different AS mechanisms, transcript isoform functions, and resistance outcomes. **a** *Arabidopsis thaliana RPS4*: Upon infection with *Pseudomonas syringae* pv. tomato DC3000 expressing effector AvrRPS4, *RPS4* produces a range of transcript isoforms through AS. Full immunity requires both the predominant regular transcript (RT) and multiple low-abundance alternative transcripts (ATs), indicating functional cooperation among isoforms. **b** *Linum usitatissimum L6*: AS of the *L6* mRNA in flax generates full-length TIR-NBS-LRR proteins and truncated variants with unique *C*-terminal extensions following recognition of the *Melampsora lini* effector A-L6. In contrast to *RPS4*, these alternative isoforms are dispensable for resistance. TIR: Toll/interleukin-1 receptor domain; NBS: Nucleotide-binding site domain; LRR: Leucine-rich repeat domain. **c** *Solanum tuberosum RB*: The *RB* mRNA is alternatively spliced into full-length and truncated NLR (NBS-LRR) isoforms, with splicing regulated by the splicing factor StCWC15 in response to the *Phytophthora infestans* effector IPI-O1. The splicing ratio fine-tunes defense, balancing immunity with growth by modulating the abundance of functional versus truncated proteins. CC: Coiled-coil domain. **d** *Triticum aestivum Pm4*: AS of the *Pm4* mRNA generates two isoforms, *Pm4b_V1* and *Pm4b_V2*, that gives rise to a protein complex activated by the effector AvrPm4 from *Blumeria graminis* f. sp. *tritici*, leading to hypersensitive resistance. S_TKc: Kinase domain with serine/threonine specificity; C2: Protein signalling motifs with a Ca^2+^-binding region; PRT_C: Phosphoribosyl transferase *C*-terminal domain. **e** *Hordeum vulgare Mla13*: AS of upstream open reading frames (uORFs) within the *Mla13* 5′ leader sequence modulates translation efficiency. This post-transcriptional regulation fine-tunes *Mla13* protein expression in barley, ensuring effective resistance against *B. graminis* f. sp. *hordei*. TLR: transcript leader region. Together, these cases exemplify the functional diversity and regulatory flexibility provided by AS in plant immune responses, ranging from isoform cooperation and modulation of resistance strength to translational control mechanisms. Created in BioRender, https://BioRender.com/e4br983

*A. thaliana RESISTANCE TO PSEUDOMONAS SYRINGAE 4* (*RPS4*) plays a pivotal role in the immune response against *P. syringae* pv. *tomato* DC3000, which expresses the effector protein AvrRPS4 (Gassmann et al. 1999; Fig. 1a). Upon effector recognition, *RPS4* undergoes AS, producing a range of transcript isoforms that includes both a predominant, regular transcript (RT) and several low-abundance alternative transcripts (ATs). The presence of both transcript types is crucial for the establishment of full immunity (Zhang and Gassmann 2003; Fig. 1a). Notably, while the RT is responsible for the core immune function, the ATs appear to collaborate with the RT to amplify or refine the immune response (Zhang and Gassmann 2003). This interplay of isoforms underscores the sophisticated nature of plant immune responses, where even low-abundance transcripts contribute to resistance by potentially enhancing or modulating the functional activity of the RT (Zhang and Gassmann 2007; Fig. 1a). These findings highlight the importance of AS in creating a network of isoform diversity that cooperates to fine-tune immune responses, emphasizing how plants use AS as a regulatory tool to ensure robust and adaptive defense mechanisms.

In flax (*Linum usitatissimum*), *FLAX RUST RESISTANCE GENE L6* (*L6*) confers resistance to the obligate biotrophic fungal pathogen *Melampsora lini*, causing flax rust (Islam and Mayo 1990; Ellis et al. 1999; Fig. 1b). Upon recognition of the pathogen effector protein A-L6, *L6* undergoes AS yielding a mix of full-length proteins consisting of TIR-NBS-LRR (Toll/interleukin-1 receptor-Nucleotide-binding site-Leucine-rich repeat) alongside truncated variants that feature unique *C*-terminal extensions (Ayliffe et al. 2008; Fig. 1b). While AS plays a pivotal role in generating isoform diversity in this context, the alternative isoforms are dispensable for resistance. In contrast to immune systems in which alternative isoforms are essential for full resistance (e.g., AS of *RPS4*), the full-length L6 protein is sufficient to trigger the immune response, and the truncated isoforms do not function in pathogen recognition or defense activation (Ayliffe et al. 2008; Fig. 1b). Hence, while AS is a common feature in plant immune responses, the functional roles of alternative transcripts can vary significantly across species, reflecting the context-specific nature of immune signalling.

In potato (*Solanum tuberosum*), *RPI-BLB1 (RB*) plays a central role in resistance against the oomycete pathogen *Phytophthora infestans*, the causative agent of late blight (Song et al. 2003; Fig. 1c). *RB* encodes a protein featuring the NBS-LRR domains, typical of plant immune receptors (Song et al. 2003; Fig. 1c). Upon pathogen attack, *RB* undergoes AS to generate both full-length and truncated isoforms of the NLR protein (Sun et al. 2024; Fig. 1c). This splicing event is regulated by the splicing factor StCWC15, which modulates the production of these isoforms in response to the effector protein, IN PLANTA INDUCED 1, VARIANT O1 (Sun et al. 2024; Fig. 1c), also known as Avrblb1 from *P. infestans* (Vleeshouwers et al. 2008). The presence of both full-length (*RB_CDS*) and truncated (*RB_IR*) isoforms allows for a delicate balance between effective immunity and the potential trade-off with plant growth. Full-length isoform *RB_CDS* encodes an active R protein and is thought to be fully functional in pathogen detection and immune activation, whereas truncated isoform *RB_IR* may serve as a regulatory mechanism to limit excessive defense responses that could impede plant growth (Sun et al. 2024; Fig. 1c). This splicing-mediated regulation ensures that the plant can mount an appropriate immune response without compromising its overall fitness, highlighting the complex role of AS in regulating immunity and plant development.

Wheat (*Triticum aestivum*) *Pm4* confers resistance to *Blumeria graminis* f. sp. *tritici*, the causal agent of wheat powdery mildew (Ullah et al. 2018; Fig. 1d). *Pm4* undergoes AS to produce two distinct isoforms, *Pm4b_V1* and *Pm4b_V2* which participate in forming a protein complex that is critical for initiating a hypersensitive response (Sánchez-Martín et al. 2021; Fig. 1d). The formation of this isoform-specific protein complex is essential for resistance, as it activates downstream signalling pathways leading to effective immune responses (Sánchez-Martín et al. 2021; Fig. 1d). This highlights the crucial role that AS plays in modulating the formation of immune complexes and influencing resistance outcomes.

In barley (*Hordeum vulgare*), *Mla13*, a gene encoding a mildew locus A protein, plays a critical role in resistance to *Blumeria graminis* f. sp. *hordei*, a pathogen that causes barley powdery mildew (Halterman et al. 2003). AS in the 5′ leader region of *Mla13*, particularly through modulation of upstream open reading frames (uORFs), fine-tunes translation efficiency (Halterman et al. 2003; Fig. 1e). Mutagenesis-based inactivation of the uORFs in the 5′-UTR indicates that these elements negatively regulate translation (Halterman and Wise 2006). Such post-transcriptional control is essential for maintaining appropriate levels of Mla13, ensuring that the immune response is not overactive or underactive to minimise host cell damage (Halterman et al. 2003; Halterman and Wise 2006; Fig. 1e). This form of translational regulation underscores the importance of AS in not only controlling transcript levels but also in modulating translation efficiency to fine-tune immune responses.

## AS events increase following abiotic stress treatment

Plants, being sessile, face challenges in unaccommodating environmental conditions and have evolved through AS to confront heat, low temperature, drought and salt stresses (Punzo et al. 2020; Lam et al. 2022). Comparative splicing analysis using RNA-seq libraries, after cold, salt, dehydration, or ABA treatment in canola (*Brassica napus*), revealed the occurrence of AS-induced events, with intron retention most prevalent (Yang L et al. 2022). Of the 357 differential AS genes identified from *B. napus*, 81 had recurred following a different source of abiotic stress (cold, salt, dehydration, or ABA treatment) (Yang L et al. 2022).

**Fig. 2:**
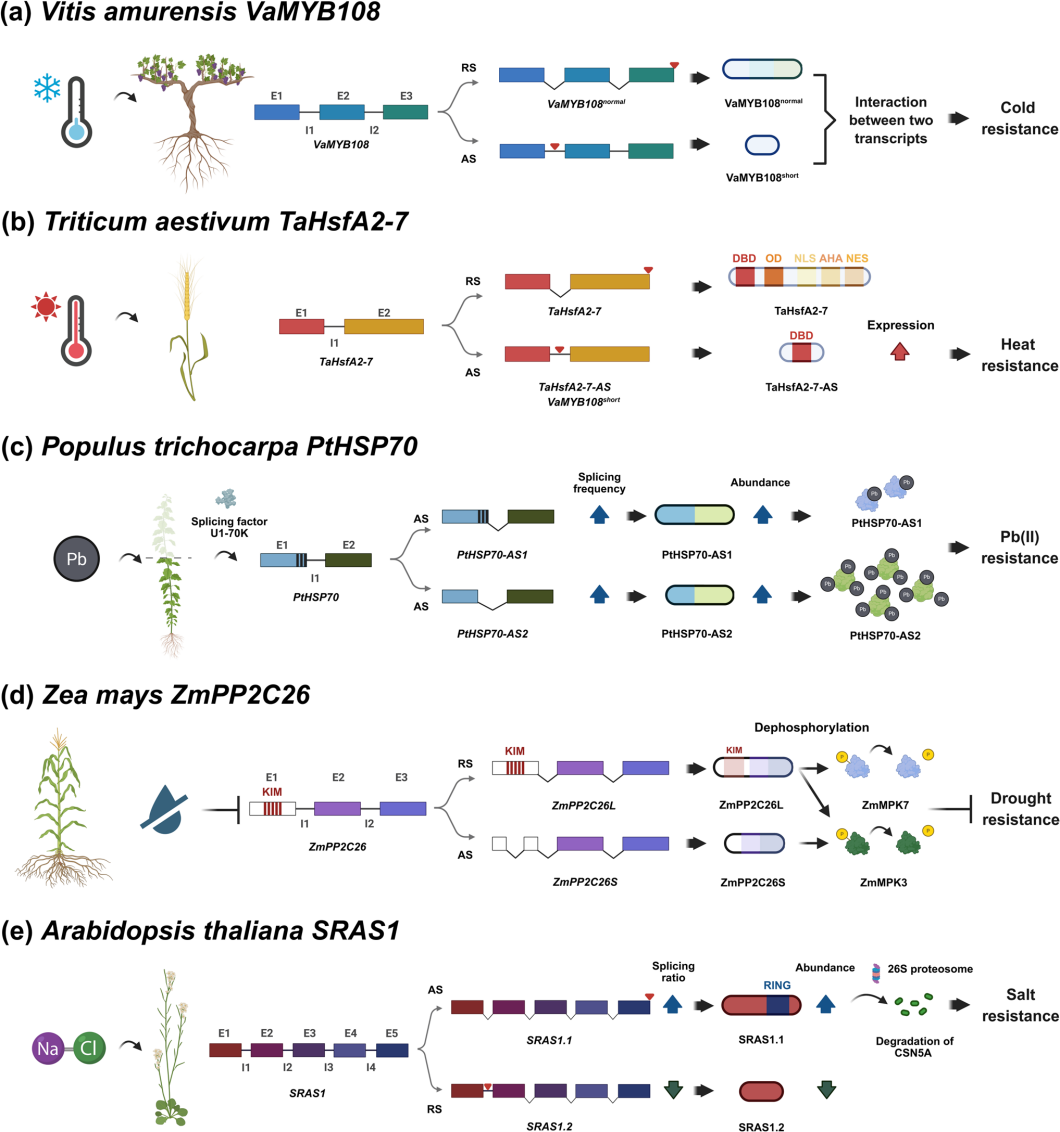
Alternative splicing diversifies the function of stress-responsive genes to enhance abiotic stress tolerance in plants. AS of key regulatory genes enables plants to generate distinct transcript isoforms with specialized functions to fine-tune their responses to various abiotic stresses. The spliced and retained introns are shown as angled and straight lines, respectively. The cryptic introns are indicated by vertically hatched boxes in the exons. E: exon; I: intron. **a**-**e** The examples span across various species and highlight different AS mechanisms, transcript isoform functions, and resistance outcomes. **a** *VaMYB108* encoding *Vitis amurensis* transcription factor (Hou et al. 2024): AS of the *VaMYB108* mRNA yields two isoforms: *VaMYB108*^*normal*^ and *VaMYB108*^*short*^, with differential exon usage. These transcripts interact functionally, and their co-expression enhances cold resistance, possibly through modulation of transcriptional responses or protein interactions during cold stress. **b** *TaHsfA2-7* encoding *Triticum aestivum* heat stress transcription factor (Ma et al. 2023): The *TaHsfA2-7* mRNA undergoes both regular splicing (RS) and AS, producing two isoforms: *TaHsfA2-7* and *TaHsfA2-7-AS*. The full-length isoform contains conserved domains including DBD (DNA-binding domain), OD (oligomerization domain), NLS (nuclear localization signal), AHA (activation domain), and NES (nuclear export signal), promoting heat resistance. The alternatively spliced isoform lacks key regulatory domains, indicating a possible role in fine-tuning the heat response. **c** *PtHSP70* encoding *Populus trichocarpa* chaperone protein (Zhu et al. 2024): Under lead (Pb^2^⁺) stress, the *PtHSP70* mRNA undergoes AS, regulated by the splicing factor U1-70 K, generating two isoforms: *PtHSP70-AS1* and *PtHSP70-AS2*. Both isoforms show increased expression under Pb stress, with PtHSP70-AS2 exhibiting a tenfold higher Pb^2^⁺-binding capacity and a stronger transcriptional response compared to PtHSP70-AS1. These isoforms are believed to enhance Pb^2^⁺ tolerance by stabilizing proteins and buffering heavy metal toxicity. It is further speculated that HSP70 may assist in exporting Pb^2^⁺ ions from plant cells, possibly through interactions with heavy metal transport pumps, contributing to cellular detoxification and Pb^2^⁺ resistance. **d** *ZmPP2C26* encoding *Zea mays* serine/threonine protein phosphatase 2C (Lu et al. 2022): The *ZmPP2C26* mRNA produces two isoforms through AS: *ZmPP2C26L* and *ZmPP2C26S*. ZmPP2C26S lacks 71 amino acids, including the MAPK interaction motif (KIM), and shows higher phosphatase activity. ZmPP2C26L interacts with and dephosphorylates both mitogen-activated protein kinases (MAPK) ZmMAPK3 and ZmMAPK7, while ZmPP2C26S only dephosphorylates ZmMAPK3. Under drought stress, the expression of both isoforms is significantly reduced, suggesting stress-regulated splicing and a role in modulating drought resistance through differential MAPK signalling. **e** *SRAS1* encoding *Arabidopsis thaliana* RING finger E3 ligase (Zhou et al. 2021): The *SRAS1* mRNA is alternatively spliced into two isoforms: SRAS1.1 and SRAS1.2. SRAS1.1, a full-length E3 ubiquitin ligase contains a RING domain, functions as an E3 ligase, and promotes degradation of CSN5A (COP9 signalosome subunit 5A) via the 26S proteasome. Meanwhile, SRAS1.2 lacks the RING domain and does not facilitate CSN5A degradation. Under normal conditions, SRAS1.2 is predominant and binds to CSN5A, stabilizing it within the COP9 signalosome complex to regulate plant growth. Salt stress increases the ratio of SRAS1.1, promoting CSN5A degradation via the 26S proteasome, adjusting stress responses and enhancing salt tolerance. Created in BioRender, https://BioRender.com/8hnhcjw

In adaptation to low temperature, single-molecule real-time (SMRT) sequencing on an extremely cold-tolerant wild grape, *Vitis amurensis*, identified 189 genes in low-temperature stress response in root from an analysis of 2958 genes producing 8797 AS events (Hou et al. 2024). Of these, the short and normal transcripts of the transcription factor VaMYB108 encoded 103 and 307 amino acid residues, respectively (Hou et al. 2024; Fig. 2a). When these isoforms were overexpressed in transgenic grapevine hairy roots, both conferred protection against cold tolerance (Hou et al. 2024; Fig. 2a). Given that the roots overexpressing either isoforms lacked significant differences before and after low temperature treatment when assayed for superoxide dismutase (SOD), peroxidase (POD), hydrogen peroxide, proline and soluble sugar content, VaMYB108 did not appear to regulate SOD, POD, hydrogen peroxide, proline and soluble sugar content to influence low temperature resistance. Although soluble sugar content in transformed hairy roots did not vary before and after low temperature treatment, both lines showed higher soluble sugar content than the vector-transformed roots after treatment, indicating that both isoforms probably influenced soluble sugar content at low temperature (Hou et al. 2024). Although both VaMYB108 protein isoforms were targeted to the nucleus, the prematurely terminated shorter protein did not display transcriptional activation activity (Hou et al. 2024).

Using full-length transcriptome sequencing, AS was detected following cold (10 °C) treatment on two cultivars of peanut (*Arachis hypogaea*), with intron retention and exon skipping being most common (Wang et al. 2024). This comparative transcriptomic analysis of *A. hypogaea* cultivars demonstrated that the cold-sensitive cultivar produced more differentially expressed genes (DEGs) while the cold-tolerant cultivar yielded greater AS events (Wang et al. 2024). Treatment carried out at 3 °C and − 3 °C for 3 h on the cold-sensitive *Populus trichocarpa* and the cold-tolerant *P. ussururiensis*, respectively (Yang et al. 2021), followed by SMRT-Seq and Illumina RNA Seq revealed AS in *P. trichocarpa* (1,261 AS events) and *P. ussururiensis* (2101 AS events) with intron retention predominant (Yang et al. 2021). Resembling *A. hypogaea* (Wang et al. 2024), the cold-tolerant *P. ussururiensis* (Yang et al. 2021) gave a higher number of AS events. Furthermore, the number of up and downregulated DEGs increased with declining temperature in almost all AS types and more genes were downregulated than upregulated in *P. ussururiensis* than *P. trichocarpa* (Yang et al. 2021).

Splicing memory occurs when a past stress event, such as non-lethal heat stress, alters the RNA splicing pattern culminating in a long-lasting change such that upon stress recurrence, the plant has already acquired an ability to face the stress (Ling et al. 2018). Heat stress which reduces productivity, triggers AS events and plants primed to non-lethal heat stress retained splicing memory from transcriptomic analysis between primed and non-primed *A. thaliana* (Ling et al. 2018). In comparison to non-primed plants, de-repression of splicing occurred in primed plants following the second heat treatment (Ling et al. 2018). Heat-responsive genes subject to AS are widespread in plants, including alfafa (*Medicago sativa*), cabbage (*Brassica oleracea*), lily (*Lilium longiflorum*), maize (*Zea mays*), poplar (*P. trichocarpa*), rice (*O. sativa*), tomato (*Solanum lycopersicum*) and wheat (*Triticum aestivum*) (Ling et al. 2021). Many heat-responsive transcription factors, such as heat shock proteins (HSPs), heat stress transcription factor A1 (HSFA1) and the dehydration-responsive element binding protein 2B (DREB2B), are liable to AS (Ling et al. 2021). For example, *TaHSFA6e* which encodes a heat shock transcription factor is alternatively spliced to yield transcripts *TaHSFA6e-II* and *TaHSFA6e-III* (Wen et al. 2023). TaHSFA6e-II lacks a 14-amino acid peptide of trytophan residues at its *C*-terminus in contrast to TaHSFA6e-III (Wen et al. 2023). The AS-derived peptide in TaHSFA6e-III forms a predicted amphipathic helix that represents a putative contact site for transcriptional activation and effectively promotes transcription of three downstream heat shock protein 70 (TaHSP70) genes, which in turn enhances thermotolerance by regulating re-initiation of translation (Wen et al. 2023). The overexpression of an alternative-spliced derivative of the heat shock transcription factor TaHsfA2-7 (designated TaHsfA2-7-AS) protected yeast and transgenic *A. thaliana* from heat stress (Ma et al. 2023; Fig. 2b). In comparison to TaHsfA2-7, TaHsfA2-7-AS is a truncated isoform possessing only a partial DNA-binding domain (Fig. 2b), and though targeted to the nucleus, it lacks transcriptional activation activity (Ma et al. 2023).

The NineTeen Complex (NTC)-related protein, NTR1, functions as an accessory during spliceosome disassembly with its expression suppressed upon heat stress in *A. thaliana* (He et al. 2023). NTR1 plays a role in regulating transcription and post-transcription of heat stress response genes, and downregulation of the NTR1 complex after heat stress caused improper splicing accompanied by toxic product accumulation (He et al. 2023). Evidence supporting NTR1 function in promoting heat stress tolerance, derived from studies using heat-susceptible *ntr1* mutants, indicated an increase in false splicing of heat shock response (*HSR*) genes and accumulation of intron retention (IR)-related AS events (He et al. 2023). After heat stress, suppression in the expression of NTR1 and NTR1-associated complex components caused rapid transcription of heat shock transcription factors (HSFs) and heat shock proteins (HSPs), followed by improper splicing (He et al. 2023).

Using Sequential Window Acquisition of all Theoretical Mass Spectra (SWATH-MS)-based proteogenomic analysis, Zhu et al. (2024) showed that lead [Pb(II)] treatment of poplar (*P. trichocarpa*), a species used in bioremediation, caused AS with non-conventional splice site usage and an accumulation of Pb(II)-responsive splicing factors (SFs) associated with Pb(II)-responsive transcription factors. In proteogenomic analysis, which utilises genomic and proteomic data to better understand the relationship between genes and proteins, the following techniques are utilised in parallel: RNA-seq, including short-read RNA sequencing (srRNA-seq) and long-read RNA sequencing (lrRNA-seq), plus SWATH-MS-based proteomics to enable a deeper interpretation of the potential genome coding capacity (Zhu et al. 2024). This approach will overcome any weak correlation between protein abundance and transcript expression and facilitate the identification of targets that may be controlled at transcript and protein levels (Zhu et al. 2024). Subsequent characterization of a gene encoding a strong Pb(II)-inducible chaperone protein, PtHSP70, revealed spliced variants (PtHSP70-AS1 and PtHSP70-AS2) of which PtHSP70-AS2, lacking in the first 105 amino acids at the* N*-terminus, demonstrated higher expression under Pb(II) stress as well as a ten-fold capacity to bind Pb(II) (Zhu et al. 2024; Fig. 2c). When the spliced isoforms were overexpressed in *A. thaliana* and poplar (*Populus deltoides* × *Populus euramericana)*, the transgenic plants were conferred Pb(II) tolerance, with PtHSP70-AS2 being more effective (Zhu et al. 2024; Fig. 2c).

In *A. thaliana*, recent findings have highlighted the role of AS of the *VEGETATIVE GROWTH TO REPRODUCTIVE GROWTH TRANSITION FACTOR 1* (*VRF1*) as a crucial regulatory mechanism that mediates the balance between abiotic stress tolerance and early flowering. Rather than altering overall gene expression, the plant fine-tunes the relative abundance of two functional splice isoforms, VRF1-AS1 and VRF1-AS3, in response to environmental cues such as drought. These isoforms competitively interact with the MITOGEN-ACTIVATED PROTEIN KINASE KINASE 1 (MKK1), inducing distinct phosphorylation events that activate separate downstream signalling pathways, one promoting stress resilience while the other triggers reproductive transition (Chen et al. 2024). This AS-mediated molecular switch highlights the broader significance of AS in dynamic environmental adaptation, serving as a source of transcript diversity while simultaneously acting as a precise post-transcriptional regulatory mechanism that modulates physiological responses and enables plants to swiftly transition between survival and reproductive strategies in fluctuating environments (Chen et al. 2024).

In maize (*Z. mays*), ZmPP2C26, encoding a serine/threonine protein phosphatase 2C (PP2C), negatively regulates drought tolerance by the dephosphorylation of mitogen-activated protein kinases, ZmMAPK3 and ZmMAPK7 (Lu et al. 2022). Following AS, *ZmPP2C26* produced two splice variants (ZmPP2C26L and ZmPP2C26S), which showed lower expression after drought stress (Lu et al. 2022; Fig. 2d). While ZmPP2C26L dephosphorylates both ZmMAPK3 and ZmMAPK7, ZmPP2C26S acts only on ZmMAPK3 (Lu et al. 2022; Fig. 2d). In comparison to ZmPP2C26L, ZmPP2C26S lacks 71 amino acids inclusive of a MAPK interaction domain, and displays higher phosphatase activity (Lu et al. 2022). Also, ZmPP2C26L, which retains a 213-bp sequence in the first exon in contrast to ZmPP2C26S (Fig. 2d), is directed to the nucleus and chloroplast, while ZmPP2C26S is targeted to the nucleus and cytosol (Lu et al. 2022). The overexpression of each splice variant in *A. thaliana* and *O. sativa* reduced drought tolerance accompanied by decreases in root length, chlorophyll content and photosynthesis (Lu et al. 2022). When ectopically expressed in transgenic *O. sativa*, ZmPP2C26L regulates protein phosphorylation predominantly in photosynthesis, while ZmPP2C26S controls proteins in carbon fixation related to photosynthesis and pyruvate metabolism (Lu et al. 2022).

## Salt stress triggers AS in plants

Soil salinization adversely impacts crop production, but plants have acquired solutions, including AS, to survive. Strategies in AS involve high-affinity potassium transporters, peptidyl-prolyl *cis*–trans isomerase, RING finger E3 ligase, and spliceosomal proteins (e.g., spliceosomal core protein, nuclear cyclophilins) and in soybean (*Glycine max*) Class II acyl-CoA-binding proteins (GmACBPs). Resembling ZmPP2C in drought stress whereby the shorter AS variant (ZmPP2C26S) lacks a protein–protein interacting domain (Lu et al. 2022), Class II GmACBP splice variants (GmACBPsv) lack the full ankyrin repeat domain essential for protein–protein interaction (Lung et al. 2022). The Class II GmACBP splice variants, derived from AS after salt treatment, showed accumulation of both salt-tolerant and salt-sensitive soybean varieties, with greater abundance in the latter (Lung et al. 2022). In salt-treated soybean, the Class II GmACBP variants can no longer interact with the protein partner, lipoxygenase (LOX) designated VLXB (Lung et al. 2022). Thus, VLXB is liberated to generate oxylipin signals and trigger the salinity response (Lung et al. 2022). Besides oxylipins, the salt-stress-associated signalling molecule phosphatidic acid (PA) was generated from membrane phospholipids mediated by Class II ACBPs in *A. thaliana* (Lung et al. 2022). The production of both lipid signals triggers the salinity response and confer protection (Lung et al. 2022). Under normal conditions, the endoplasmic reticulum (ER)-anchored Class II GmACBPs bind with fatty acid (FA) ligands (linoleoyl- and linolenoyl-CoAs) to mediate protein–protein interaction with LOXs, thereby suppressing LOX activity by sequestering them at the ER membrane (Lung et al. 2022). High salinity induces the dissociation of the GmACBP-LOX complex in two ways: PA signals generated compete with FAs for the same lipid-binding site on GmACBPs or AS produces GmACBP, which compete with the native proteins for the same ligands. FA release weakens the GmACBP-LOX protein interaction, liberating LOX to generate oxylipin signals (Lung et al. 2022). Furthermore, transgenic *A. thaliana* overexpressing the AS variants (GmACBPsv) were salt-tolerant in comparison to the salt-sensitive *A. thaliana* overexpressing the full-length Class II GmACBPs (Lung et al. 2022).

In the desert poplar (*P. euphratica*), high-affinity potassium transporters (HKTs) maintain K^+^ ion homeostasis, and AS causing a deletion at the first exon of the transcript (*PeuHKT*) yields *PeuHKT1:3a* (Lv et al. 2024). The overexpression of *PeuHKT1:3a* in transgenic *A. thaliana* and poplar (*P. alba* var. *pyramidalis*) enhanced salt tolerance because 84 amino acids, including a Ser residue within the first pore-loop domain, critical in promoting Na^+^ uptake, was absent in this splice variant (Lv et al. 2024). This variant showed a higher affinity for K^+^, causing a higher K^+^/ Na^+^ ratio and improved salt tolerance (Lv et al. 2024). Salt treatment also induced AS in foxtail millet (*Panicum italicum*), with 2078 AS events identified in RNA-seq analysis (Zhang Y et al. 2024). For example, *SiCYP19* encoding peptidyl-prolyl *cis*–trans isomerase gave two splice variants (*SiCYP19-a* and *SiCYP19-b*) that enhanced growth when overexpressed in yeast upon salt stress (Zhang Y et al. 2024). Comparison of the amino acid sequences of the splice variants revealed that *SiCYP19-a* contains a 15-bp insert that encodes five additional amino acids at the* N*-terminus. Given that this could potentially affect protein targeting, subcellular localization studies were conducted and showed that the AS-derived protein, SiCYP19-a, was localized to the nucleus in contrast to mitochondria-bound SiCYP19-b. Furthermore, *Panicum italicum* overexpressing *SiCYP19-b* became salt-tolerant and gained ability to scavenge reactive oxygen species and increase proline content (Zhang Y et al. 2024).

Global profiling of transcriptome RNA-seq datasets of Chinese liquorice (*Glycyrrhiza uralensis*) indicated that AS, with exon skipping being the most common, was significantly induced in response to salt treatment (Yao et al. 2024). A comparison between the underground and above-ground tissue of salt-stressed *G. uralensis* suggested that AS was more prevalent underground while exon skipping accounted for 28–29% and 30–34% of AS-responsive events in underground and above-ground tissue, respectively (Yao et al. 2024). Also, underground tissue exhibited greater AS pattern alterations (Yao et al. 2024). Furthermore, the Kyoto Encyclopedia of Genes and Genomes (KEGG) enrichment analysis indicated an increase in AS-associated pathways (for RNA transport, mRNA surveillance and the spliceosome), implicating splicing regulation (Yao et al. 2024). Genes related to mRNA surveillance, known to function in the detection of abnormal mRNAs and in enabling maintenance of accurate gene expression, were also been reported to prevail in soybean roots under drought stress (Song et al. 2020). In tomato (*S. lycopersicum*) root, high-throughput RNA-seq analysis disclosed that AS occurs in the early response to salt stress, with 3709 genes identified to undergo AS, with exon skipping predominant (Gan et al. 2024). The differentially alternatively spliced genes included serine/threonine protein kinase, pentatricopeptide repeat (PPR)-containing protein and E3 ubiquitin-protein ligase (Gan et al. 2024). DEGs identified were associated with splicing and spliceosome assembly, advocating AS in *S. lycopersicum* roots (Gan et al. 2024).

In Najran wheat (*Triticum aestivum*), transcriptome analysis revealed that AS occurred in 22.5% (32,268 AS events) and 23.1% (31,941 AS events) of expressed genes in roots and shoots, respectively, with the use of 3' alternative splice site prevalent (Alyahya and Taybi 2024). Furthermore, salt treatment caused tissue-specific AS responses with 82% differentially expressed in either root or shoots implying that the strategies applied to counteract salt stress differ between these organs (Alyahya and Taybi 2024). Moreover, only 74 genes were reported to undergo differential splicing in both organs (Alyahya and Taybi 2024). In another monocot, rice (*O. sativa*), AS events following salt stress were documented using a software tool to quantify and visualize Variations of Splicing in Population (VASP) intended for genome-wide association studies (GWAS) (Yu et al. 2021). From short-read RNA-seq datasets, VASP was used to quantify splicing variants and detect from diverse genotypes, variations in AS post-salt treatment, culminating in the eventual identification of 764 significant genotype-specific splicing events (Yu et al. 2021).

Alternative splicing of an *A. thaliana* salt-responsive gene *SALT RESPONSIVE* *ALTERNATIVELY SPLICED1* (*SRAS1*) encoding a RING finger E3 ligase, yielded two antagonistic variants, *SRAS1.1* and *SRAS1.2*, of which *SRAS1.1* showed greater accumulation (Zhou et al. 2021; Fig. 2e). Comparison of the variants revealed that as a result of intron retention, *SRAS1*.*2* encodes a truncated isoform (59-amino acid peptide) lacking the *C*-terminal RING domain while *SRAS1*.*1* encodes a normal SRAS protein (221-amino acid peptide) inclusive of the RING domain (Zhou et al. 2021; Fig. 2e). In contrast to SRAS1.1, the severely truncated SRAS1.2 is unable to function as an active E3 ligase, lacks ability to regulate salt stress signalling and mediate salt-responsive gene expression and cannot interact with CSN5A, a putative protein partner of SRAS1.1 (Zhou et al. 2021). Furthermore, SRAS1.1, an essential subunit of CSN5 that forms part of the COP9 signalosome (CSN), was subsequently demonstrated to promote CSN5A degradation by the 26S proteasome under salt stress (Zhou et al. 2021). SRAS1.2 thus lacks the biological activity to protect against salt stress in comparison to SRAS1.1 (Zhou et al. 2021; Fig. 2e). Consequently, transgenic* A. thaliana* SRAS1.1-overexpressors were salt tolerant (Fig. 2e) while SRAS1.2-overexpressors were salt sensitive (Zhou et al. 2021). The target for SRAS1 was proven to be COP9 signalosome 5A (CSN5A), which is essential in regulating stress and development (Zhou et al. 2021). SRAS1.1 promoted CSN5A degradation by the 26S proteasome, while SRAS1.2 competes with SRAS1.1. and protected CSN5A (Zhou et al. 2021; Fig. 2e). Hence, AS modulates E3 ligase function during salt stress (Zhou et al. 2021).

## Other spliceosomal-related proteins in stress protection

Besides the spliceosome-associated proteins, OsFKB20-1b (Jung et al. 2023), LSM4 (Agrofoglio et al. 2024) and NTR1 (He et al. 2023), other proteins that play a role in AS-related stress protection include the *A. thaliana* spliceosomal core protein SmEb. Transcriptome analysis indicated that proper splicing maintenance by SmEb is important in conferring salt tolerance after salinity treatment, as SmEb affects the splicing of many genes, and *smeb* mutants are deficient in AS (Hong et al. 2023). For example, *RCD1* (*RADICAL-INDUCED CELL DEATH1*), which regulates plant stress responses, yields two splice variants, *RCD1.1* and *RCD1.2* (Hong et al. 2023). SmEb maintains a ratio of the RCD1 splicing variants (RCD1.1: RCD1.2) to regulate the salt stress response as RCD1.1 (not RCD1.2), localized in nuclear speckles, can interact with stress regulators (Hong et al. 2023). The overexpression of *RCD1.1* in transgenic *smeb* mutant *A. thaliana* protects against oxidative damage and conferred salt tolerance, by partially recovering the increased salt sensitivity in *smeb*. In contrast, *RCD1.2,* which retains the fourth intron, produces a truncated peptide that is deprived of the domain that interacts with stress regulators (Hong et al. 2023). Hence, SmEb regulates *RCD1* post-transcriptionally in salt protection through controlling the ratio of these two splice variants (Hong et al. 2023).

Besides NTR (He et al. 2023), accessory proteins in spliceosome complexes, such as nuclear cyclophilins (CYPs), are known to function in *A. thaliana* pre-mRNA splicing (Jo et al. 2022). Following heat stress in seed germination, CYP-18 was demonstrated to be essential for the removal of retention-prone introns by activation of the dephosphorylation splicing factor PRP18 (Jo et al. 2022). CYP18-1 plays a role in spliceosome restructuring involving PRP18 dephosphorylation to facilitate acclimatization during heat stress (Jo et al. 2022).

Other factors that regulate abiotic stress responses through RNA splicing include the SR proteins (Jia et al. 2023), such as the *O. sativa* OsACR106 from the subfamily SC (Alhabsi et al. 2024). Its mutant (*scr106*) was not only hypersensitive to salt, ABA, and low-temperature stress but also showed shorter shoot and root formation (Alhabsi et al. 2024). The OsSCR106 splicing factor regulates the alternative 3’-splice site and ensures accurate pre-mRNA splicing in abiotic responses (Alhabsi et al. 2024). Although sweet potato (*Ipomoea batatas*) *SR* genes generally indicate conservation to the *A. thaliana* and *O. sativa* homologues (e.g., in possessing 24 genes), some of the introns in *SR* genes, such as *IbSR31* had atypical borders including GG-CA, AG-GC, AG-TG and GA-GT instead of the conserved GT-AG borders (Chen et al. 2022). In addition, heat rather than salt or drought treatment more substantially affected AS of *SR* genes in *I. batatas* (Chen et al. 2022). This conclusion, drawn from RT-PCR analysis, points out that following heat stress, nine *SR* genes showed an increase in the alternatively-spliced variants over the controls, while only four produced more alternatively-spliced variants in either drought or salt treatment (Chen et al. 2022).

## Methodologies available in studying AS in stress adaptation

It has been suggested that the apparently lower percentage of plant genes estimated to undergo AS in comparison to human genes, stem from incomplete analysis of plant genomes (Bedre et al. 2019). However, advances in technology, such as full-length transcriptome sequencing (e.g. long-read SMRT), high-throughput RNA sequencing, and direct RNA-sequencing platforms, as well as bioinformatics have facilitated effective characterization of the transcriptome, indicating that AS occurs more frequently than previously envisioned (Bedre et al. 2019; Petrillo et al. 2020). Table 1 summarises these research methods to investigate AS in plants, with their advantages and disadvantages listed. The occurrence of AS can be initially validated using reverse transcriptase-polymerase chain reaction (RT-PCR) and DNA sequencing to identify the alternatively-spliced transcripts and quantitative RT-PCR can be used to quantify transcripts in the presence and absence of the respective stress (Bedre et al. 2019; Table 1). The relative cost of these methods, scope of application, and examples in which they have been utilised to decipher the molecular mechanism or key factors in AS regulations are summarized in Table 1. Liu et al. (2024) proposed the use of a combination of genetic studies, quantitative techniques and high-throughput omics techniques to investigate AS regulation in plants. These techniques include RT-PCR and absolute quantification of spliced variants by QuantAS (Song et al. 2023). The latter incorporates quantitative PCR with digital PCR, followed by the analysis and integration of data derived from these two techniques to quantify all the spliced isoforms by use of specific primers and absolute quantification technology (Liu et al. 2024; Song et al. 2023). The use of distinct probes will enable the simultaneous detection of variants and eliminate any difficulties in isoform identification (Liu et al. 2024; Song et al. 2023). Also, a multigenomics approach has been suggested to elucidate the transcriptome by high-throughput sequencing and the proteome by mass spectrometry to better understand AS (Liu et al. 2024). Liu et al. (2024) proposed that techniques such as SMART-seq2 can be utilised for single-cell transcriptome sequencing while SMART-seq3 will be more sensitive for the detection of the number of variants. Furthermore, SWATH-MS-based proteogenomic analysis can be applicable in studies, as reported in lead [Pb(II)] treatment of *P. trichocarpa* (Zhu et al. 2024; Table 1).

**Table 1:** Comparison of research methods for investigations on alternative splicing in plants

Research method	Advantages	Disadvantages	Scope of application	Relative cost #	Applications in references
Direct RNA-seq platforms	cDNA synthesis	Requires bioinformatics	Detects novel RNA	+ + + + + +	Yao et al. (2024)
	and RNA seq in native	tools/expertise; higher error	modification		
	form not necessary;	rates lower throughput;			
	accuracy in RNA	greater RNA needed;			
	modification detection	high costs			
SWATH-MS-based	Label-free quantification;	Requires spectral libraries,		+ + + + + +	Zhu et al. (2024)
proteogenomic analysis	spectral library can be	specialized software,			
	reused	computational resources			
		for data analysis; poor			
		sensitivity in detection			
		of low-abundance proteins			
Full-length transcriptome	Entire sequences of	Low throughput;	1-20 kb reads possible;	+ + + + +	Hou et al. (2024)
seq (e.g. long-read SMRT)	RNA transcripts	more complex	e.g., whole genome seq;		Yang et al. (2021)
whole exome seq					Wang et al. (2024)
Bioinformatics for processing	Process massive	Algorithm-dependent;	Hastens drug discovery,	+ + + +	Liu et al. (2024)
RNA-seq data	sets quickly	costly development and	analysis of complex		
		maintenance of databases	biological processes		
High-throughput RNA seq	Smaller transcript	Harder identification	Comprehensive	+ + +	Liu et al. (2024)
	fragments sequenced;	with the shorter reads;	transcriptome study on		
	discovery of novel	needs reference genome,	gene expression, for		
	transcripts	bioinformatic analysis	drug discovery		
Next generation seq	Quick, cheap identification	Limited in discovery,	DNA sequence analysis	+ + +	Wang et al. (2020)
	of alternatively-spliced	more relevant to			
	transcripts	elucidation of DNA			
qRT-PCR	Quantify transcripts of	Unsuitable for discovery	Quantification	+ +	Zhang et al. (2024a, b)
	known/specific genes	of new genes; limited	of RNA in gene		Wang et al. (2024)
	in presence/absence	multiplexing	expression analysis		
	the respective stress;				
	relatively cheap				
RT-PCR	RNA as template;	Unsuitable for discovery	mRNA quantification;	+	Zhang et al. (2024a, b)
	high-sensitivity yields quick results; relatively cheap	of new genes; limited multiplexing	analysis of differential gene expression; genotyping		Wang et al. (2024

*seq* Sequencing, *SMRT* Single-molecule real-time, *qRT-PCR* Quantitative reverse transcriptase-polymerase chain reaction, *RT-PCR* Reverse transcriptase-polymerase chain reaction

^#^ Relative cost with “ + ” the cheapest

A comprehensive database of plant-specific AS events in response to stress is now available for the model plants, *A. thaliana* and rice, with conservation in AS patterns upon stress treatments affirmed (PlaASDB; http://zzdlab.com/PlaASDB/ASDB/index.html; Guo et al. 2023). To construct this database, 3255 RNA-seq data under biotic and abiotic stress treatments from both model plants were collected and subsequent AS detection as well as gene expression analysis were conducted (Guo et al. 2023). Specifically, a total of 2703 RNA-Seq data sets, including 2280 abiotic and 423 biotic stress experiments, were obtained from *A. thaliana*, and a total of 552 RNA-Seq data sets, including 410 abiotic and 142 biotic stress experiments were obtained from rice and analysed (http://zzdlab.com/PlaASDB/ASDB/index.html). The samples were subject to an exon-based AS detection tool to locate the major AS events (IR, ES, A5SS, and A3SS). A comparison of the AS patterns between these plants revealed limited overlap between differentially spliced genes and DEGs after stress treatments (e.g., drought, salt, wounding, osmotic stress, salicylic acid, ABA), indicative that AS and the regulation of gene expression assume independent roles in stress responses (Guo et al. 2023). Also, it was reported that these two model plants share conserved AS patterns during stress. The database represents a promising resource to acquire a better understanding of the AS landscape under various stresses because it provides integrated information on AS and gene expression in stress responses and as well as large-scale comparative analyses, allowing researchers to comprehend the regulatory mechanisms in AS (Guo et al. 2023). Information from the website (http://zzdlab.com/PlaASDB/ASDB/index.html), indicates that PlaASDB users can initiate a search by gene ID or name, followed by the types of AS events involving the gene when subject to different stress conditions. Also, users can view each transcript for information on AS, including the type, length, location, and the average Percent-Splice In (PSI) value. The availability of a short description of the co-expressed genes and corresponding links to other databases, PLaASDB allows users access to potential gene function. Furthermore, co-expression networks under abiotic and biotic stresses and information on the functional roles in abiotic and biotic stresses are available inclusive of AS events and co-expression partners. This database is expected to expedite investigations on AS and functional genomics of stress events in plants.

**Fig. 3:**
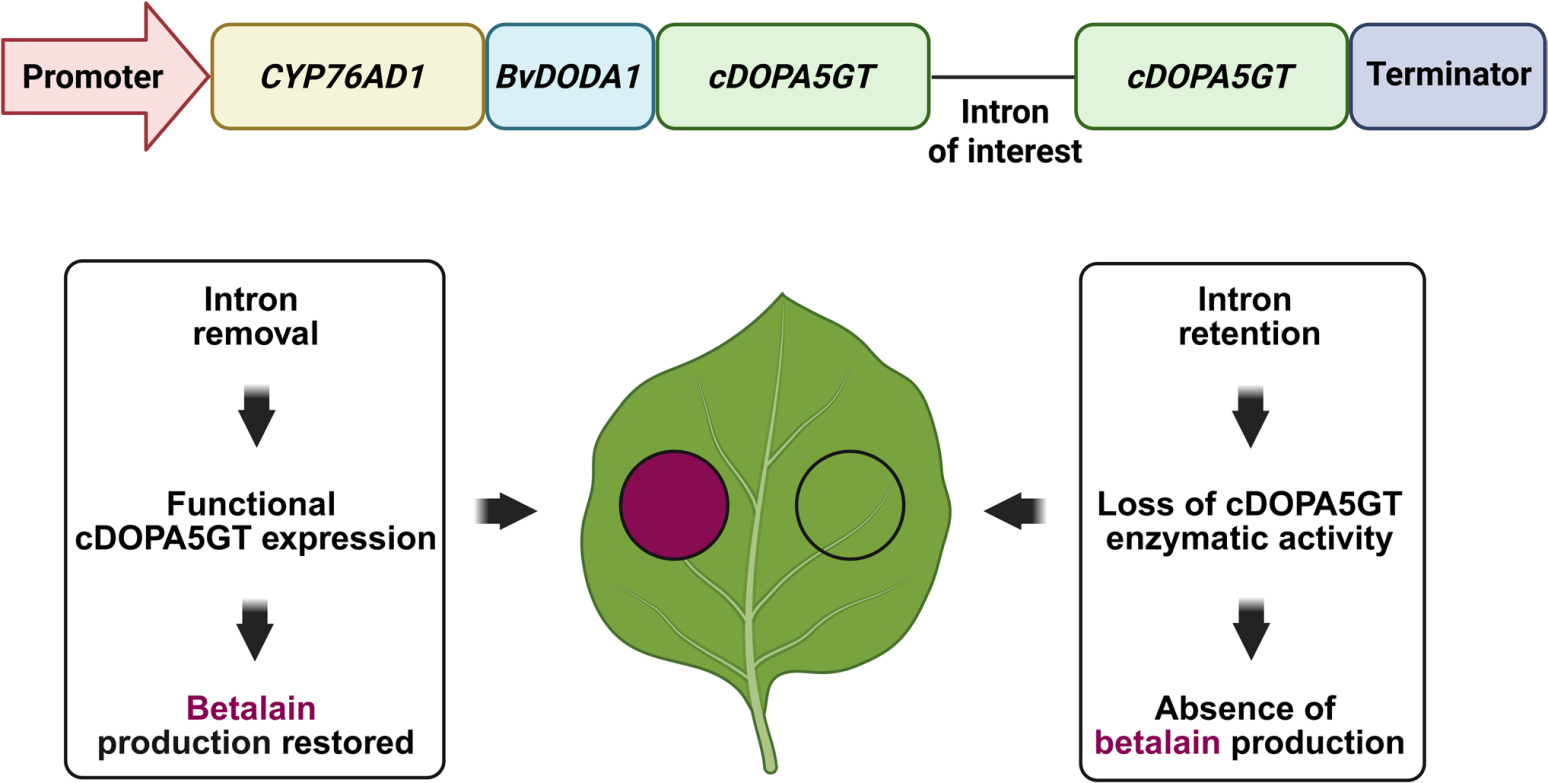
Visual assay for testing intron splicing efficiency *in planta* using *RUBY* reporter gene. A synthetic construct can be developed to assess whether a candidate intron is correctly spliced in plant cells (Prasad et al. 2024). The construct includes a constitutive promoter driving the expression of three betalain biosynthetic genes: *CYP76AD1*, *BvDODA1*, and *cDOPA5GT*, with the intron of interest inserted within the coding region of *cDOPA5GT*. Left panel: Upon successful intron removal, a functional cDOPA5GT enzyme is produced, enabling full betalain biosynthesis, leading to visible red pigmentation in infiltrated *N. benthamiana* leaves. Right panel: If the intron is retained, cDOPA5GT remains non-functional, resulting in loss of enzymatic activity and the absence of betalain accumulation. This approach serves as a simple, visual reporter system for intron splicing efficiency, enabling rapid functional testing of intronic sequences in vivo. Created in BioRender, https://BioRender.com/2pzsgmx

In another recent development, a method utilizing an encoded visual splicing reporter RUBY was devised to non-invasively track in vivo pre-mRNA splicing (Prasad et al. 2024). Synthetic *Ruby*, a fusion of three genes for betaine biosynthesis (*CYP76AD1*, *BvDODA1,* and *cDOPA5GT*), converts tyrosine to visible red betalain, that can be sighted by the naked eye (He et al. 2020). The splicing reporter was generated by the introduction of an *A. thaliana* RSZ32 (encoding a serine/arginine-rich splicing factor) wild-type constitutively-spliced intron (*Ruby-Intron*) or a 5’ and 3’ splice site mutated intron (*Ruby*_*m*_*Intron*_*m*_) into *cDOPA5GT* encoding the glucosylytransferase component in *Ruby* (Prasad et al. 2024). Intron removal from *Ruby-Intron* produces a functional transcript for cDOPA5GT resulting in a red colour (Prasad et al. 2024; Fig. 3). In contrast, retention of the intron with *Ruby*_*m*_*Intron*_*m*_ will cause premature transcript termination and the red colour will be absent (Prasad et al. 2024; Fig. 3). The use of RUBY, which does not require expensive substrates or specialized equipment to visualize fluorescence or light emission will conveniently allow researchers to unravel AS through real-time tracking *in planta* (Prasad et al. 2024).

The successful implementation of the *RSZ32* intron serves as a proof of concept for the ability of *RUBY* to report on intron splicing. Importantly, this framework can be readily extended to study the splicing behaviour of other introns, making it a valuable tool for screening intron splicing efficiency, identifying *cis*-regulatory elements, or evaluating mutant alleles and splicing factors in diverse plant backgrounds. The *RUBY* system is compatible with both transient expression and stable transformation platforms and has been validated in multiple species, including *A. thaliana*, *Nicotiana benthamiana*, *Plukenetia volubilis*, *O. sativa*, and *Z. mays* (He et al. 2020; Yu et al. 2023; Pramanik et al. 2024). It holds particular promise for forward genetic screens aimed at identifying *trans*-acting splicing regulators and for studying how splicing is modulated under abiotic and biotic stresses (Alhabsi et al. 2025).

However, the effectiveness of the *RUBY* reporter depends on sufficient betalain pigment accumulation, which is influenced by the expression level of the construct. Low expression leads to subthreshold betalain accumulation, resulting in weak or absent colouration and a high risk of false-negative results (Yu et al. 2023). Thus, strong constitutive promoters are recommended for transgenic applications to ensure a robust signal output. Additionally, environmental and physiological factors such as pH, light, temperature, and enzyme stability could be affecting the betalain production and may contribute to variability in reporter expression among transgenic lines due to post-transcriptional regulation (Pérez-Ramírez et al. 2015; Khan 2016; Khidr et al. 2017). Hence, to fully harness its potential for high-throughput screening and long-term genetic studies, further improvements in the sensitivity and stability of *RUBY* expression will be essential.

## Conclusions

AS is now known to be widespread in plants, including major food crops, such as rice (*O. sativa*; Dong et al. 2018), wheat (*T. aestivum*; Wen et al. 2023) and maize (*Z. mays*; Chen et al. 2018). In *O. sativa*, AS can control phenotypic variation affecting leaf, panicle and height, as well as agronomic traits that are relevant to breeding (Doll 2024; Zhang et al. 2024a). From the resource *PastDB* (https://pastdb.crg.eu/wiki/Main_Page) for *A. thaliana* which represents an atlas of AS profiles across tissues, development and environment, including biotic and abiotic treatments, it appears that in comparison to animals, *A. thaliana* utilises AS disproportionally under stress, with exon skipping overrepresented (Martin et al. 2021). Given the relationship between AS and environmental stress, AS appears to be closely related to plant adaptation and evolution (Ling et al. 2019). However, investigations on genome-wide AS using transcriptomic datasets of Arabidopsis (*A. thaliana*), soybean (*G. max*), tomato (*S. lycopersicum*) and wild tobacco (*Nicotiana. attenuata*), to represent a wide range of eudicots, species-specific clustering of AS pattern revealed low conservation of AS among species and comparison between divergent species and closely-related species indicated high variability in AS amongst plants (Ling et al. 2019).

Recent knowledge acquired from AS events in plants arising from biotic and abiotic stresses pertaining to the molecular mechanisms will be useful in designing crops that can be potentially protected against phytopathogens and environmental stressors. Climate change, causing drought, a rise in soil salinity and seawater overflow from flooding will require the production of salinity-tolerant crops. Global warming will require AS-related strategies to produce heat resilient varieties. A better molecular understanding of the cellular sensors and signalling pathways related to heat stress is expected to improve agriculture and enhance food security (Calixto 2025). In summary, Fig. 1 illustrates examples of how AS-derived variants of *R* genes contribute to immune responses against diverse pathogens, including bacteria, fungi, oomycetes, and rusts across multiple plant species, while Fig. 2 highlights the potential role of AS-derived variants in protection against abiotic stresses such as cold, heat, lead [Pb(II)], drought, and salt. AS-transcripts have been verified to confer stress tolerance in at least one plant species (Fig. 2) and their potential remains to be assessed in others.

## Data Availability

Not applicable.

## Abbreviations

AS: Alternative splicing
DEG: Differentially expressed gene

## References

[CR1] Agrofoglio YC, Iglesias MJ, Perez-Santángelo S, de Leone MJ, Koester T, Catalá R, Salinas J, Yanovsky MJ, Staiger D, Mateos JL (2024) Arginine methylation of SM-LIKE PROTEIN 4 antagonistically affects alternative splicing during Arabidopsis stress responses. Plant Cell 36:2219–2237. 10.1093/plcell/koae051 38518124 10.1093/plcell/koae051 PMC11132874

[CR2] Alhabsi A, Butt H, Kirschner GK, Blilou I, Mahfouz MM (2024) SCR106 splicing factor modulates abiotic stress responses by maintaining RNA splicing in rice. J Exp Bot 75:802–818. 10.1093/jxb/erad433 37924151 10.1093/jxb/erad433 PMC10837019

[CR3] Alhabsi A, Ling Y, Crespi M, Reddy ASN, Mahfouz M (2025) Alternative splicing dynamics in plant adaptive responses to stress. Annu Rev Plant Biol 76:687–717. 10.1146/annurev-arplant-083123-090055 39952682 10.1146/annurev-arplant-083123-090055

[CR4] Alyahya N, Taybi T (2024) Transcriptome-wide characterization of alternative splicing regulation in Najran wheat (*Triticum aestivum*) under salt stress. Curr Plant Biol 38:100334. 10.1016/j.cpb.2024.100334

[CR5] Ayliffe MA, Frost DV, Finnegan EJ, Lawrance GJ, Anderson PA, Ellis JG (2008) Analysis of alternative transcripts of the fax *L6* rust resistance gene. Plant J 17:287–292. 10.1046/j.1365-313X.1999.00377.x 10.1046/j.1365-313x.1999.00377.x 10097386

[CR6] Bedre R, Irigoyen S, Petrillo E, Mandadi KK (2019) New era in plant alternative splicing analysis enabled by advances in high-throughput sequencing (HTS) technologies. Front Plant Sci 10:740. 10.3389/fpls.2019.00740 31231413 10.3389/fpls.2019.00740 PMC6558643

[CR7] Braunschweig U, Barbosa-Morais NL, Pan Q, Nachman EN, Alipanahi B, Gonatopoulos-Pournatzis T, Frey B, Irimia M, Blencowe BJ (2014) Widespread intron retention in mammals functionally tunes transcriptomes. Genome Res 24:1774–1786. 10.1101/gr.177790.114 25258385 10.1101/gr.177790.114 PMC4216919

[CR8] Calixto CPG (2025) Molecular aspects of heat stress sensing in land plants. Plant J 121:e70069. 10.1111/tpj.70069 40085177 10.1111/tpj.70069 PMC11908636

[CR9] Chen Q, Han Y, Liu H, Wang X, Sun J, Zhao B, Li W, Tian J, Liang Y, Yan J, Yang X, Tian F (2018) Genome-wide association analyses reveal the importance of alternative splicing in diversifying gene function and regulating phenotypic variation in maize. Plant Cell 30:1404–1423. 10.1105/tpc.18.00109 29967286 10.1105/tpc.18.00109 PMC6096592

[CR10] Chen S, Mo Y, Zhang Y, Zhu H, Ling Y (2022) Insights into sweet potato SR proteins: from evolution to species-specific expression and alternative splicing. Planta 256:72. 10.1007/s00425-022-03965-5 36083517 10.1007/s00425-022-03965-5

[CR11] Chen MX, Tian Y, Zhu FY, Fan T, Yan HX, Sun PC, Li M, Hou XX, Lin P, Song YC, Yang X, Lu CM, Yang JC, Reddy ASN, Zhang JH, Liu YG (2024) Alternative splicing of VRF1 acts as a molecular switch to regulate stress-induced early flowering. Cell Rep 43:114918. 10.1016/j.celrep.2024.114918 39488828 10.1016/j.celrep.2024.114918

[CR13] Deng D, Gu L, Liu C, Lu T, Lu T, Lu Z, Cui P, Pei Y, Wang B, Hu S, Cao X (2010) Arginine methylation mediated by the *Arabidopsis* homolog of PRMT5 is essential for proper pre-mRNA splicing. Proc Natl Acad Sci USA 107:19114–19119. 10.1073/pnas.1009669107 20956294 10.1073/pnas.1009669107 PMC2973915

[CR14] Doll NM (2024) New insights into alternative splicing in rice using population-level transcriptomics. Plant Cell 36:4276–4277. 10.1093/plcell/koae214 39041484 10.1093/plcell/koae214 PMC11449057

[CR15] Dong C, He F, Berkowitz O, Liu J, Cao P, Tang M, Shi H, Wang W, Li Q, Shen Z, James Whelan J, Zheng L (2018) Alternative splicing plays a critical role in maintaining mineral nutrient homeostasis in rice (*Oryza sativa*). Plant Cell 30:2267–2285. 10.1105/tpc.18.00051 30254029 10.1105/tpc.18.00051 PMC6241280

[CR16] Ellis JG, Lawrence GJ, Luck JE, Dodds PN (1999) Identification of regions in alleles of the flax rust resistance gene *L* that determine differences in gene-for-gene specificity. Plant Cell 11:495–506. 10.1105/tpc.11.3.495 10072407 10.1105/tpc.11.3.495 PMC144189

[CR17] Gan J, Qiu Y, Tao Y, Zhang L, Okita TW, Yan Y, Tian L (2024) RNA-seq analysis reveals transcriptome reprogramming and alternative splicing during early response to salt stress in tomato root. Front Plant Sci 15:1394223. 10.3389/fpls.2024.1394223 38966147 10.3389/fpls.2024.1394223 PMC11222332

[CR18] Gassmann W, Hinsch ME, Staskawicz BJ (1999) The Arabidopsis *RPS4* bacterial-resistance gene is a member of the TIR-NBS-LRR family of disease-resistance genes. Plant J 20:265–277. 10.1046/j.1365-313x.1999.t01-1-00600.x 10571887 10.1046/j.1365-313x.1999.t01-1-00600.x

[CR19] Guo X, Wang T, Jiang L, Qi H, Zhang Z (2023) PlaASDB: a comprehensive database of plant alternative splicing events in response to stress. BMC Biol 23:225. 10.1186/s12870-023-04234-7 10.1186/s12870-023-04234-7 PMC10134664 37106367

[CR20] Halterman DA, Wise RP (2006) Upstream open reading frames of the barley *Mla13* powdery mildew resistance gene function co-operatively to down-regulate translation. Mol Plant Pathol 7:167–176. 10.1111/j.1364-3703.2006.00329.x 20507437 10.1111/j.1364-3703.2006.00329.x

[CR21] Halterman DA, Wei F, Wise RP (2003) Powdery mildew-induced *Mla* mRNAs are alternatively spliced and contain multiple upstream open reading frames. Plant Physiol 131:558–567. 10.1104/pp.014407 12586880 10.1104/pp.014407 PMC166832

[CR22] He Y, Zhang T, Sun H, Zhan H, Zhao Y (2020) A reporter for noninvasively monitoring gene expression and plant transformation. Hortic Res 7:152. 10.1038/s41438-020-00390-1 33024566 10.1038/s41438-020-00390-1 PMC7502077

[CR23] He L, Wu Q, Jin Y, Fan Y, Shi H, Wang Y, Yang W (2023) *NTR1* is involved in heat stress tolerance through mediating expression regulation and alternative splicing of heat stress genes in *Arabidopsis*. Front Plant Sci 13:1082511. 10.3389/fpls.2022.1082511 36704159 10.3389/fpls.2022.1082511 PMC9871932

[CR24] Hong Y, Gao Y, Pang J, Shi H, Li T, Meng H, Kong D, Chen Y, Zhu JK, Wang Z (2023) The Sm core protein SmEb regulates salt stress responses through maintaining proper splicing of *RCD1* pre-mRNA in *Arabidopsis*. J Integr Plant Biol 65:1383–1393. 10.1111/jipb.13457 36661041 10.1111/jipb.13457

[CR25] Hou Y, Li Q, Zhou H, Kafle S, Li W, Tan L, Liang J, Meng L, Xin H (2024) Smrt sequencing of a full-length transcriptome reveals cold induced alternative splicing in *Vitis amurensis* root. Plant Physiol Biochem 213:108863. 10.1016/j.plaphy.2024.108863 38917739 10.1016/j.plaphy.2024.108863

[CR26] Islam MR, Mayo GME (1990) A compendium on host genes in flax conferring resistance to flax rust. Plant Breed 104:89–100. 10.1111/j.1439-0523.1990.tb00409.x

[CR27] Jabre I, Reddy ASN, Kalyna M, Chaudhary S, Khokhar W, Byrne LJ, Wilson CM, Syed NH (2019) Does co-transcriptional regulation of alternative splicing mediate plant stress responses? Nucleic Acids Res 47:2716–2726. 10.1093/nar/gkz121 30793202 10.1093/nar/gkz121 PMC6451118

[CR28] Jia ZC, Das D, Zhang Y, Fernie AR, Liu YG, Chen M, Zhang J (2023) Plant serine/arginine-rich proteins: versatile players in RNA processing. Planta 257:109. 10.1007/s00425-023-04132-0 37145304 10.1007/s00425-023-04132-0

[CR30] Jo SH, Park HJ, Lee A, Jung H, Park JM, Kwon SY, Kim HS, Lee HJ, Kim YS, Jung C, Cho HS (2022) The Arabidopsis cyclophilin CYP18-1 facilitates PRP18 dephosphorylation and the splicing of introns retained under heat stress. Plant Cell 34:2383–2403. 10.1093/plcell/koac084 35262729 10.1093/plcell/koac084 PMC9134067

[CR32] Jung H, Park HJ, Jo SH, Lee A, Lee HJ, Kim HS, Jung C, Cho HS (2023) Nuclear OsFKBP20-1b maintains SR34 stability and promotes the splicing of retained introns upon ABA exposure in rice. New Phytol 238:2476–2494. 10.1111/nph.18892 36942934 10.1111/nph.18892

[CR33] Khan MI (2016) Stabilization of betalains: a review. Food Chem 197:1280–1285. 10.1016/j.foodchem.2015.11.043 26675869 10.1016/j.foodchem.2015.11.043

[CR34] Khidr YA, Flachowsky H, Haselmair-Gosch C, Thill J, Miosic S, Hanke MV, Stich K, Halbwirth H (2017) Evaluation of a *MdMYB10/GFP43* fusion gene for its suitability to act as reporter gene in promoter studies in *Fragaria vesca* L. ‘Rügen.’ Plant Cell Tissue Organ Cult 130:345–356. 10.1007/s11240-017-1229-0 28781398 10.1007/s11240-017-1229-0 PMC5515962

[CR35] Kim N, Lee J, Yeom SI, Kang NJ, Kang WH (2024) The landscape of abiotic and biotic stress-responsive splice variants with deep RNA-seq datasets in hot pepper. Sci Data 11:381. 10.1038/s41597-024-03239-7 38615136 10.1038/s41597-024-03239-7 PMC11016105

[CR36] Lam PY, Wang L, Lo C, Zhu FY (2022) Alternative splicing and its roles in plant metabolism. Int J Mol Sci 23:7355. 10.3390/ijms23137355 35806361 10.3390/ijms23137355 PMC9266299

[CR37] Ling Y, Serrano N, Gao G, Atia M, Mokhtar M, Woo YH, Bazin J, Veluchamy A, Benhamed M, Crespi M, Gehring C, Reddy ASN, Mahfouz MM (2018) Thermopriming triggers splicing memory in Arabidopsis. J Exp Bot 69:2659–2675. 10.1093/jxb/ery062 29474581 10.1093/jxb/ery062 PMC5920379

[CR38] Ling Z, Brockmöller T, Baldwin IT, Xu S (2019) Evolution of alternative splicing in eudicots. Front Plant Sci 10:707. 10.3389/fpls.2019.00707 31244865 10.3389/fpls.2019.00707 PMC6581728

[CR39] Ling Y, Mahfouz MM, Zhou S (2021) Pre-mRNA alternative splicing as a modulator for heat stress response in plants. Trends Plant Sci 26:1153–1170. 10.1016/j.tplants.2021.07.008 34334317 10.1016/j.tplants.2021.07.008

[CR40] Liu XX, Guo QH, Xu W-B, Liu P, Yan K (2022) Rapid regulation of alternative splicing in response to environmental stresses. Front Plant Sci 13:832177. 10.3389/fpls.2022.832177 35310672 10.3389/fpls.2022.832177 PMC8931528

[CR41] Liu Y, Do S, Huynh H, Li J-X, Liu Y-G, Du Z-Y, Chen MX (2024) Importance of pre-mRNA splicing and its study tools in plants. Adv Biotechnol 2:4. 10.1007/s44307-024-00009-9 10.1007/s44307-024-00009-9 PMC11740881 39883322

[CR42] Lu F, Li W, Peng Y, Cao Y, Qu J, Sun F, Yang Q, Lu Y, Zhang X, Zheng L, Fu F, Yu H (2022) *ZmPP2C26* alternative splicing variants negatively regulate drought tolerance in maize. Front Plant Sci 13:851531. 10.3389/fpls.2022.851531 35463404 10.3389/fpls.2022.851531 PMC9024303

[CR43] Lung SC, Lai SH, Wang H, Zhang X, Liu A, Guo ZH, Lam HM, Chye ML (2022) Oxylipin signaling in salt-stressed soybean is modulated by ligand-dependent interaction of Class II acyl-CoA-binding proteins with lipoxygenase. Plant Cell 34:1117–1143. 10.1093/plcell/koab306 34919703 10.1093/plcell/koab306 PMC8894927

[CR44] Lv J, Zhou F, Wei Q, Long X, Tian W, Zhai J, Wang J, Zhang Q, Wan D (2024) An alternative 3’ splice site of *PeuHKT1;3* improves the response to salt stress through enhancing affinity to K^+^ in *Populus*. Plant Physiol Biochem 212:108776. 10.1016/j.plaphy.2024.108776 38843683 10.1016/j.plaphy.2024.108776

[CR45] Ma Z, Li M, Zhang H, Zhao B, Liu Z, Duan S, Meng X, Li G, Guo X (2023) Alternative splicing of *TaHsfA2-7* is involved in the improvement of thermotolerance in wheat. Int J Mol Sci 24:1014. 10.3390/ijms24021014 36674529 10.3390/ijms24021014 PMC9861123

[CR46] Mandadi KK, Petrillo E, Dubrovina AS, Kiselev KV (2023) Editorial: regulation of alternative splicing in plant stress responses. Front Plant Sci 13:1120961. 10.3389/fpls.2022.1120961 36733599 10.3389/fpls.2022.1120961 PMC9888408

[CR48] Marasco LE, Kornblihtt AR (2023) The physiology of alternative splicing. Nat Rev Mol Cell Biol 24:242–254. 10.1038/s41580-022-00545-z 36229538 10.1038/s41580-022-00545-z

[CR49] Martín C, Márquez Y, Mantica F, Duque P, Irimia M (2021) Alternative splicing landscapes in *Arabidopsis thaliana* across tissues and stress conditions highlight major functional differences with animals. Genome Biol 22:35. 10.1186/s13059-020-02258-y 33446251 10.1186/s13059-020-02258-y PMC7807721

[CR50] Pérez-Ramírez E, Lima E, Guzmán A (2015) Natural betalains supported on γ-alumina: a wide family of stable pigments. Dye Pigment 120:161–168. 10.1016/j.dyepig.2015.03.040

[CR51] Petrillo E, Kalyna M, Mandadi KK, Tu SL, Simpson CG (2020) Editorial: alternative splicing regulation in plants. Front Plant Sci 11:913. 10.3389/fpls.2020.00913 32733504 10.3389/fpls.2020.00913 PMC7363971

[CR52] Pramanik D, Lee K, Wang K (2024) A simple and efficient method for betalain quantification in RUBY-expressing plant samples. Front Plant Sci 15:1449409. 10.3389/fpls.2024.1449409 39359623 10.3389/fpls.2024.1449409 PMC11445021

[CR53] Prasad KVSK, Cheema A, Scanlon W, Matthews A, Sharifova S, Huq E, Reddy ASN (2024) A simple method to visualize pre-mRNA splicing with the naked eye using a genetically encoded visual splicing reporter. Plant Physiol 196:726–730 39056536 10.1093/plphys/kiae396 PMC11444279

[CR54] Punzo P, Grillo S, Batelli G (2020) Alternative splicing in plant abiotic stress responses. Biochem Soc Trans 48:2117–2126. 10.1042/BST20200281 32869832 10.1042/BST20200281

[CR55] Sánchez-Martín J, Widrig V, Herren G, Wicker T, Zbinden H, Gronnier J, Spörri L, Praz CR, Heuberger M, Kolodziej MC, Isaksson J, Steuernagel B, Karafiátová M, Doležel J, Zipfel C, Keller B (2021) Wheat *Pm4* resistance to powdery mildew is controlled by alternative splice variants encoding chimeric proteins. Nat Plants 7:327–341. 10.1038/s41477-021-00869-2 33707738 10.1038/s41477-021-00869-2 PMC7610370

[CR56] Song J, Bradeen JM, Naess SK, Raasch JA, Wielgus SM, Haberlach GT, Liu J, Kuang H, Austin-Phillips S, Buell CB, Helgeson JP, Jiang J (2003) Gene *RB* cloned from *Solanum bulbocastanum* confers broad spectrum resistance to potato late blight. Proc Natl Acad Sci USA 100:9128–9133. 10.1073/pnas.1533501100 12872003 10.1073/pnas.1533501100 PMC170883

[CR57] Song YC, Chen MX, Zhang KL, Reddy ASN, Cao FL, Zhu FY (2023) QuantAS: a comprehensive pipeline to study alternative splicing by absolute quantification of splice isoforms. New Phytol 240:928–939. 10.1111/nph.19193 37596706 10.1111/nph.19193

[CR82] Song L, Pan Z, Chen L, Dai Y, Wan J, Ye H, Nguyen HT, Zhang G, Chen H (2020) Analysis of whole transcriptome RNA-seq data reveals many alternative splicing events in soybean roots under drought stress conditions. Genes 11:1520. 10.3390/genes11121520 33352659 10.3390/genes11121520 PMC7765832

[CR58] Sun B, Huang J, Kong L, Gao C, Zhao F, Shen J, Wang T, Li K, Wang L, Wang Y, Halterman DA, Dong S (2024) Alternative splicing of a potato disease resistance gene maintains homeostasis between growth and immunity. Plant Cell 36:3729–3750. 10.1093/plcell/koae189 38941447 10.1093/plcell/koae189 PMC11371151

[CR59] Sybilska E, Daszkowska-Golec A (2023) Alternative splicing in ABA signaling during seed germination. Front Plant Sci 14:1144990. 10.3389/fpls.2023.1144990 37008485 10.3389/fpls.2023.1144990 PMC10060653

[CR60] Szakonyi D, Duque P (2018) Alternative splicing as a regulator of early plant development. Front Plant Sci 9:1174. 10.3389/fpls.2018.01174 30158945 10.3389/fpls.2018.01174 PMC6104592

[CR61] Ullah KN, Li N, Shen T, Wang P, Tang W, Ma S, Zhang Z, Jia H, Kong Z, Ma Z (2018) Fine mapping of powdery mildew resistance gene *Pm4e* in bread wheat (*Triticum aestivum* L.). Planta 248:1319–1328. 10.1007/s00425-018-2990-y 30128601 10.1007/s00425-018-2990-y

[CR62] Vleeshouwers VG, Rietman H, Krenek P, Champouret N, Young C, Oh SK, Wang M, Bouwmeester K, Vosman B, Visser RG, Jacobsen E, Govers F, Kamoun S, Van der Vossen EA (2008) Effector genomics accelerates discovery and functional profiling of potato disease resistance and *Phytophthora infestans* avirulence genes. PLoS ONE 3:e2875. 10.1371/journal.pone.0002875 18682852 10.1371/journal.pone.0002875 PMC2483939

[CR63] Wang Z, Ji H, Yuan B, Wang S, Su C, Yao B, Zhao H, Li X (2015) ABA signalling is fine-tuned by antagonistic HAB1 variants. Nat Commun 6:8138. 10.1038/ncomms9138 26419884 10.1038/ncomms9138

[CR64] Wang L, Chen M, Zhu F, Fan T, Zhang J, Lo C (2020) Alternative splicing is a *Sorghum bicolor* defense response to fungal infection. Planta 251:14. 10.1007/s00425-019-03309-w 10.1007/s00425-019-03309-w 31776670

[CR65] Wang X, Liu Y, Ouyang L, Yao R, Yu T, Yan L, Chen Y, Huai D, Zhou X, Wang Z, Kang Y, Wang Q, Jiang H, Lei Y, Liao B (2024) Full-length transcriptome sequencing provides insights into alternative splicing under cold stress in peanut. Front Plant Sci 15:1362277. 10.3389/fpls.2024.1362277 38516669 10.3389/fpls.2024.1362277 PMC10954824

[CR66] Wen J, Qin Z, Sun L, Zhang Y, Wang D, Peng H, Yao Y, Hu Z, Ni Z, Sun Q, Xin M (2023) Alternative splicing of *TaHSFA6e* modulates heat shock protein-mediated translational regulation in response to heat stress in wheat. New Phytol 239:2235–2247. 10.1111/nph.19100 37403528 10.1111/nph.19100

[CR67] Xue X, Jiao F, Xu H, Jiao Q, Zhang X, Zhang Y, Du S, Xi M, Wang A, Chen J, Wang M (2021) The role of RNA-binding protein, microRNA and alternative splicing in seed germination: a field need to be discovered. BMC Plant Biol 21:194. 10.1186/s12870-021-02966-y 33882821 10.1186/s12870-021-02966-y PMC8061022

[CR68] Yang J, Lv W, Shao L, Fu Y, Liu H, Yang C, Chen A, Xie X, Wang Z, Li C (2021) Pacbio and Illumina RNA sequencing identify alternative splicing events in response to cold stress in two poplar species. Front Plant Sci 12:737004. 10.3389/fpls.2021.737004 34691113 10.3389/fpls.2021.737004 PMC8529222

[CR69] Yang L, Yang L, Zhao C, Liu J, Tong C, Zhang Y, Cheng X, Jiang H, Shen J, Xie M, Liu S (2022a) Differential alternative splicing genes and isoform co-expression networks of *Brassica napus* under multiple abiotic stresses. Front Plant Sci 13:1009998. 10.3389/fpls.2022.1009998 36311064 10.3389/fpls.2022.1009998 PMC9608124

[CR71] Yang X, Jia Z, Pu Q, Tian Y, Zhu F, Liu Y (2022b) ABA mediates plant development and abiotic stress via alternative splicing. Int J Mol Sci 23:3796. 10.3390/ijms23073796 35409156 10.3390/ijms23073796 PMC8998868

[CR72] Yao H, Li G, Gao Z, Guo F, Feng J, Xiao G, Shen H, Li H (2024) Alternative splicing responses to salt stress in *Glycyrrhiza uralensis* revealed by global profiling of transcriptome RNA-seq datasets. Front Genet 15:1397502. 10.3389/fgene.2024.1397502 39045328 10.3389/fgene.2024.1397502 PMC11263197

[CR73] Yao Q, Duan R, Feng Y, Duan D (2025) Alternative splicing analysis of stress tolerance to Al and flg22 in *Vitis quinquangularis*. Planta 261:139. 10.1007/s00425-025-04713-1 40366460 10.1007/s00425-025-04713-1

[CR74] Yu H, Du Q, Campbell M, Yu B, Walia H, Zhang C (2021) Genome-wide discovery of natural variation in pre-mRNA splicing and prioritising causal alternative splicing to salt stress response in rice. New Phytol 230:1273–1287. 10.1111/nph.17189 33453070 10.1111/nph.17189 PMC8048671

[CR75] Yu J, Deng S, Huang H, Mo J, Xu ZF, Wang Y (2023) Exploring the potential applications of the noninvasive reporter gene *RUBY* in plant genetic transformation. Forests 14:637. 10.3390/f14030637

[CR76] Zhang XC, Gassmann W (2003) *RPS4*-mediated disease resistance requires the combined presence of *RPS4* transcripts with full-length and truncated open reading frames. Plant Cell 15:2333–2342. 10.1105/tpc.013474 14523247 10.1105/tpc.013474 PMC197299

[CR77] Zhang XC, Gassmann W (2007) Alternative splicing and mRNA levels of the disease resistance gene *RPS4* are induced during defense responses. Plant Physiol 145:1577–1587. 10.1104/pp.107.108720 17951452 10.1104/pp.107.108720 PMC2151689

[CR78] Zhang H, Chen W, Zhu D, Zhang B, Xu Q, Shi C, He H, Dai X, Li Y, He W, Lv Y, Yang L, Cao X, Cui Y, Leng Y, Wei H, Liu X, Zhang B, Wang X, Guo M, Zhang Z, Li X, Liu C, Yuan Q, Wang T, Yu X, Qian H, Zhang Q, Chen D, Hu G, Qian Q, Shang L (2024a) Population-level exploration of alternative splicing and its unique role in controlling agronomic traits of rice. Plant Cell 36:4372–4387. 10.1093/plcell/koae181 38916914 10.1093/plcell/koae181 PMC11449091

[CR79] Zhang Y, Chen Z, Tian H, Wu Y, Kong Y, Wang X, Sui N (2024b) Alternative splicing plays a crucial role in the salt tolerance of foxtail millet. J Agric Food Chem 72:10814–10827. 10.1021/acs.jafc.4c00809 38710027 10.1021/acs.jafc.4c00809

[CR80] Zhou Y, Li XH, Guo QH, Liu P, Li Y, Wu CA, Yang GD, Huang JG, Zhang SZ, Zheng CC, Yan K (2021) Salt responsive alternative splicing of a RING finger E3 ligase modulates the salt stress tolerance by fine-tuning the balance of COP9 signalosome subunit 5A. PLoS Genet 17:e1009898. 10.1371/journal.pgen.1009898 34784357 10.1371/journal.pgen.1009898 PMC8631661

[CR81] Zhu FY, Chen X, Song YC, Lam LPY, Tobimatsu Y, Gao B, Chen MX, Cao FL (2024) SWATH-MS-based proteogenomic analysis reveals the involvement of alternative splicing in poplar upon lead stress. Genome Res 33:371–385. 10.1101/gr.277473.122 10.1101/gr.277473.122 PMC10078296 36963844

